# Structural and metabolic changes in rhizophores of the Cerrado species *Chrysolaena obovata* (Less.) Dematt. as influenced by drought and re-watering

**DOI:** 10.3389/fpls.2015.00721

**Published:** 2015-09-17

**Authors:** Paola M. A. Garcia, Adriana H. Hayashi, Emerson A. Silva, Rita de Cássia L. Figueiredo-Ribeiro, Maria A. M. Carvalho

**Affiliations:** ^1^Núcleo de Pesquisa em Fisiologia e Bioquímica, Instituto de BotânicaSão Paulo, Brazil; ^2^Programa de Pós-Graduação em Biodiversidade Vegetal e Meio Ambiente, Instituto de BotânicaSão Paulo, Brazil; ^3^Núcleo de Pesquisa em Anatomia, Instituto de BotânicaSão Paulo, Brazil

**Keywords:** fructans, inulin sphero-crystals, proline, underground organs, water deficit

## Abstract

The high fructan contents in underground organs of Cerrado species, high water solubility, and fast metabolism of these compounds highlight their role as carbon storage and as an adaptive feature in plants under drought. In this study, we showed that anatomical structure, in association with soluble compounds and metabolism of inulin-type fructans were modified in rhizophores of *Crysolaena obovata* submitted to water suppression and recovery after re-watering. Plants were subjected to daily watering (control), suppression of watering for 22 days (water suppression) and suppression of watering followed by re-watering after 10 days (re-watered). Plants were collected at time 0 and after 3, 7, 10, 12, 17, and 22 days of treatment. In addition to changes in fructan metabolism, high proline content was detected in drought stressed plants, contributing to osmoregulation and recovery after water status reestablishment. Under water suppression, total inulin was reduced from approx. 60 to 40%, mainly due to exohydrolase activity. Concurrently, the activity of fructosyltransferases promoted the production of short chain inulin, which could contribute to the increase in osmotic potential. After re-watering, most parameters analyzed were similar to those of control plants, indicating the resumption of regular metabolism, after water absorption. Inulin sphero-crystals accumulated in parenchymatic cells of the cortex, vascular tissues and pith were reduced under drought and accompanied anatomical changes, starting from day 10. At 22 days of drought, the cortical and vascular tissues were collapsed, and inulin sphero-crystals and inulin content were reduced. The localization of inulin sphero-crystals in vascular tissues of *C. obovata*, as well as the decrease of total inulin and the increase in oligo:polysaccharide ratio in water stressed plants is consistent with the role of fructans in protecting plants against drought.

## Introduction

Water is the most important and abundant natural resource, but also the most limiting environmental factor during the annual growth cycle of plants. Low water availability in the soil limits the productivity of natural ecosystems and can lead to water stress. As the reduction in water availability progresses, tolerant plants gradually adjust their metabolism to cope with this condition (Chaves et al., 2002, 2003).

Water stress affects many physiological and biochemical processes, resulting in metabolic changes that involve carbohydrates and other compatible solutes that are known to contribute to osmotic adjustment (Bajii et al., 2001). Several compounds are synthesized and/or mobilized under water deficit, allowing plants to maintain the osmotic adjustment and protecting cell membranes and macromolecules. Among them are the inorganic ions, amino acids, such as proline and glutamate, sucrose and its soluble derivatives, including fructans. As widely shown, these compounds contribute to keep the water status of the cell, preventing dehydration and cell damage caused by formation of reactive oxygen species (Hoekstra et al., 2001; Peshev et al., 2013). Consensus on the integral roles of proline accumulation on stress adaptation remains still controversial (Verbruggen and Hermans, 2007; Kavi Kishor and Sreenivasulu, 2013), and evidences suggest that it plays more than one function serving as osmoticum, source of carbon and nitrogen and regulatory signal at the same time (Hare and Cress, 1997).

Fructans are sucrose-based linear or branched oligo- or polymers of fructose, often containing one terminal glucose molecule, widespread in prokaryotes—fungi and algae—and in 15% of flowering plants (Hendry, 1993). Based on the glycosidic linkage between the fructose units, they are divided into different classes: inulins, occurring mainly in eudicots (*Asteraceae*) contain exclusively (2,1) linkages; levans, present in grasses, contain mainly (2,6) linkages; graminans, branched type fructans also occurring in grasses, contain both linkage types; and the neo-inulin and neo-levan types, occurring in monocots such as *Asparagus* and *Allium* (Carvalho et al., 2007).

The distribution of fructan molecule size in plant tissues also varies widely. These variations as well as changes in total amounts have been well described in different phenological phases or developmental stages in Cerrado species (Carvalho et al., 2007). Synthesis of plant fructans requires a variety of fructan biosynthetic enzymes, the fructosyltransferases (FTs), each with their own preferential donor and acceptor substrates. According to the SST/FFT model established for inulin-type fructans in Jerusalem artichoke (Edelman and Jefford, 1968), the enzyme sucrose:sucrose 1-fructosyltransferase (1-SST; EC 2.4.1.99) catalyses the production of the trisaccharide 1-kestose and glucose from sucrose. Further chain elongation is achieved by fructan:fructan 1-fructosyltransferase (1-FFT; EC 2.4.1.100). Breakdown is accomplished by fructan 1-exohydrolase (1-FEH; EC 3.2.1.80), releasing terminal fructosyl units and using water as acceptor. During the biosynthesis and breakdown of other fructan types, containing internal glucose units or (2,6) linkages, other fructosyltransferases such as fructan:fructan 6G-fructosyltransferase (6G-FFT) and sucrose:fructan 6-fructosyltransferase (6-SFT), or fructan 6-exohydrolase (6-FEH) and fructan 6&1-exohydrolase (6&1-FEH) are involved (Livingston et al., 2009).

The presence of fructans in *Asteraceae* from the Cerrado has been widely reported and was associated with the seasonal growth exhibited by the Cerrado flora as well as with low temperatures and reduced rainfall prevailing in winter (Carvalho et al., 2007; Garcia et al., 2011). In *Vernonia herbacea* (currently *Chrysolaena obovata*), variation in fructan contents and metabolism have been reported during the phenological cycle (Carvalho and Dietrich, 1993; Portes and Carvalho, 2006). Fructan mobilization by 1-FEH, concomitant with a decline in 1-SST activity, was detected during regrowth following excision of aerial organs (Asega and Carvalho, 2004) and changes in fructans and enzymes in plants submitted to water suppression were also observed (Garcia et al., 2011), demonstrating their participation in the mechanism of osmoregulation.

The presence of inulin sphero-crystals visualized under polarized light in the cortical parenchyma cells of the underground reserve organs of *Asteraceae* of the Cerrado was previously reported (Tertuliano and Figueiredo-Ribeiro, 1993). Additionally, fructans were localized outside cells in tuberous roots of *Campuloclinium chlorolepis* by histochemical and ultrastructural analyses (Vilhalva et al., 2011) and inside and outside cells of different tissues of underground organs in several *Asteraceae* species from the Cerrado and rocky fields in Brazil (Vilhalva and Appezzato-da-Glória, 2006a,b; Hayashi and Appezzato-da-Glória, 2007; Appezzato-da-Glória et al., 2008; Appezzato-da-Glória and Cury, 2011; Oliveira et al., 2013; Bombo et al., 2014; Joaquim et al., 2014; Silva et al., 2014, 2015). All these studies reported the presence of fructans in such organs, and pointed them as an adaptive feature to environmental constrains.

In the present study, we confirm the role of inulin-type fructans and proline in drought tolerance and recovery from stress, showing for the first time by an experimental approach, structural changes in response to water suppression in *Chrysolaena obovata*. Our results extend the knowledge on fructan localization in reserve organs and present evidences associating it with protective functions in plants.

## Materials and methods

### Plant material and experimental design

Plants of *Chrysolaena obovata* (Less.) M. Dematt. were obtained by means of vegetative propagation from rhizophore fragments as described in Carvalho et al. (1998), using plants collected in a cerrado area in Mogi Guaçu, SP, Brazil (22°18′S, 47°11′W). After 2 months, plants were individually transferred to 3 L pots containing cerrado soil and cultivated under glasshouse conditions. The experiment was conducted with 18-month-old plants in the vegetative stage. Mean air temperature was 23°C (maximum of 30°C and minimum of 11°C) and mean relative air humidity was 84% (maximum of 95% and minimum of 70%). During the cultivation period water was provided until pot saturation, aiming to elevate soil moisture close to field capacity in pots. Plants were separated into three groups and treated as follows: (i) daily watering—control (DW), (ii) water suppression for 22 days (WS), and (iii) water suppression for 10 days and re-watering from day 10 on (RW). Plants were collected at days zero, 3, 7, 10, 12, 17, and 22. Time of re-watering was determined by the wilting aspect of the leaves. At each sampling day, plants were submitted to measurements of fresh and dry mass of shoots and rhizophores, soil moisture, and soil and leaf water potential. Rhizophore samples were weighed and frozen in liquid nitrogen for enzyme and carbohydrate analyses. The experiment was carried out in a completely randomized design and all measurements and extractions were done in triplicate, each one corresponding to three plants.

### Soil moisture, water content, and water potential

Soil moisture was determined by gravimetric method (Blake, 1965) and soil water potential (Ψ_wsoil_) was measured using a dew point psychrometer (Decagon WP4). Water content (%) of aerial organs and rhizophores were determined using the equation WC = (FM – DM)/FM × 100, where FM is fresh mass and DM is dry mass. Leaf water potential (Ψ_wleaf_) was measured using a Scholander type pressure bomb at pre-dawn (05:00–06:00 a.m.) and rhizophore water potential (Ψ_wrhiz_) was measured in the cell sap using a dew point psychrometer model HR-33T (Wescor, Logan-UTAH).

### Light and scanning electron microscopy

For the anatomical and ultrastructural analyses, fragments of the proximal regions of the rhizophores (Portes and Carvalho, 2006) were collected at time zero (control plants) and at 10, 12, and 22 days from WS plants, and at 12 and 22 days from RW plants (re-watered at day 10).

Samples of rhizophores were fixed in FAA (formaldehyde, acetic acid, and 70% ethanol, 1:1:18, v/v) (Johansen, 1940) under vacuum. For light microscopy, samples were dehydrated in a graded ethanol series and embedded in plastic resin (Leica Historesin, Heidelberg, Germany). Sections (7–10 μm thick) were cut on a rotary microtome (Olympus CUT 4055, Waldorf, Germany), and stained with toluidine blue O (Sakai, 1973) in phosphate-citrate buffer (McIlvaine, 1921), pH 4.5. To identify inulin-type fructans, samples were fixed in 70% ethanol and sectioned freehand. Inulin crystals were visualized under polarized light and their presence was confirmed by a treatment with thymol-sulphuric acid reagent (Johansen, 1940). Permanent slides were mounted in synthetic resin Entellan (Merck, Darmstadt, Germany). Micrographs were taken with a Leica DM LB microscope (Leica, Wetzlar, Germany) equipped with a Leica DC 300F camera (Leica, Wetzlar, Germany).

For scanning electron microscopy, samples were dehydrated in a graded ethanol series and critical point-dried with CO_2_ (CPD 030 Balzers, Fürstentum, Lichtenstein) (Horridge and Tamm, 1969), mounted on aluminum stubs, coated with gold for 210 seconds (SCD 050 Balzers, Fürstentum, Liechtenstein) and examined under a scanning electron microscope (Philips XL Series XL 20, Eindhmoven, the Netherlands) at 10 kV.

### Soluble carbohydrate and proline analyses

Fructans were extracted from freeze-dried samples of rhizophores (500 mg) as previously established (Carvalho et al., 1998) and modified as follows. Tissues were boiled in 80% aqueous ethanol for 3 min for enzyme denaturation. The mixture was ground and the homogenate was kept in a water bath at 80°C for 15 min and centrifuged at 700 *g* for 15 min. This procedure was repeated twice. The residue was submitted to water extraction twice at 60°C for 30 min and filtered in cotton tissue. The ethanol and aqueous extracts were pooled, concentrated, constituting the total fructan extract. Following, it was fractionated by the addition of three volumes of ethanol, originating the fructo-oligo (mono and disaccharides, and DP ≤ 25 fructans) and fructo–polysaccharide (DP between 10 and 50) fractions after centrifugation at 2900 *g*. Free and combined fructose were measured in the two fractions by a ketose-specific modification of the anthrone reaction (Jermyn, 1956), using fructose as standard, while reducing sugars were measured in the fructo-oligosaccharide fraction, using glucose as standard (Somogyi, 1945). Carbohydrates in the oligosaccharide fraction were analyzed by HPAEC/PAD, using a 2 × 250 mm CarboPac PA-1 column on an ICS 3000 Dionex System (USA), according to Asega et al. (2011). Proline was extracted from lyophilized samples (500 mg) of rhizophores and leaves with different water contents as measured in tissues along the experiment. After extraction, proline was quantified according to Bates et al. (1973), using L-proline as standard. Results are expressed in μmol g^−1^ DM.

### Enzyme extraction and assays

Frozen samples of rhizophores (10 g) were ground in liquid nitrogen and homogenized in 0.05 M McIlvaine buffer (pH 5.5) (1:1, w/v), containing 2 mM EDTA, 2 mM β-mercaptoethanol, 5 mM ascorbic acid and 10% PVPP (w/w), as described by Asega and Carvalho (2004). Proteins precipitated with (NH_4_)_2_SO_4_ to a final saturation of 80% were re-suspended in a ratio of *ca*. 10 g fresh mass equivalent per mL in extraction buffer and desalted by centrifugation in Bio-Gel P-6 DG (BioRad, USA) equilibrated with the same buffer. The desalted protein extract was used for protein measurements (Bradford, 1976), and enzymatic assays and activity determination (Portes and Carvalho, 2006). Incubation times were 30 min for 1-FEH, 1 h for 1-SST and 1-FFT and 4 h for invertase.

### Statistical analyses

Data were analyzed by One-Way ANOVA method, using the statistical package SISVAR 4.0 (Ferreira, 2011), and significance between means was tested by the least significant difference (LSD) procedure. Relationships between Ψ_wsoil_, WC soil, Ψ_wleaf_, Ψ_wrhiz_, and the biochemical parameters were evaluated using Pearson's correlation analyses calculated with the means of each parameter. Correlations were tested for significance by Student *t-test*.

## Results

### Soil moisture, water content, and water potential

**Figure 1:**
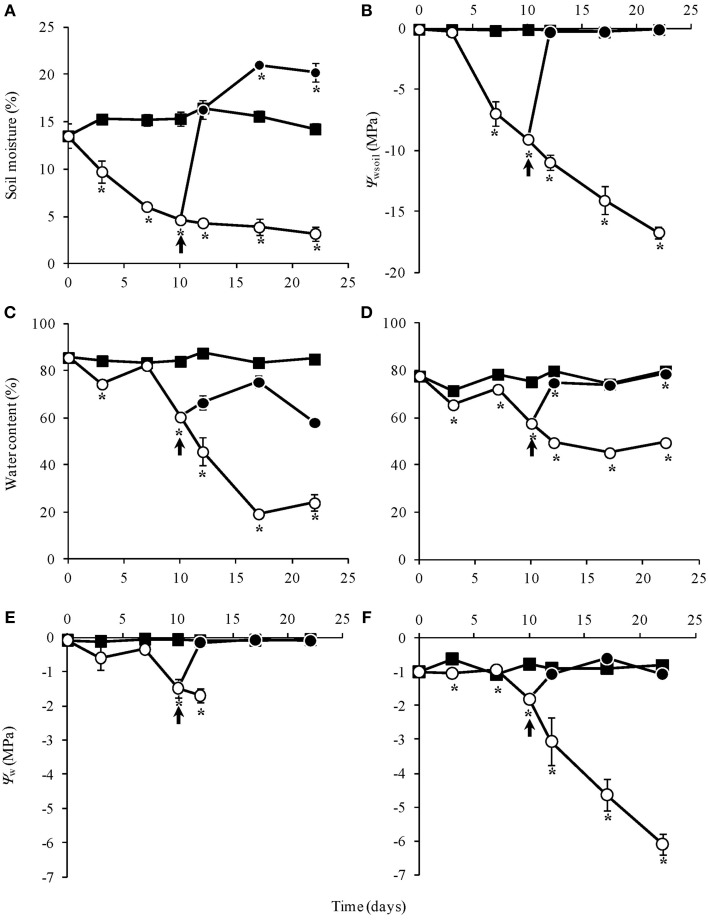
**Soil moisture (%) (A), soil water potential (Ψ_wsoil_- MPa) (B), water content (%) of aerial organs (C) and rhizophores (D), water potential (Ψ_w_- MPa) of aerial organs (E), and rhizophores (F) of *Chrysolaena obovata* submitted to daily watering, control (■); water suppression, WS (○); and re-watering, RW (●)**. Arrows indicate re-watering at day 10. Values are means ± SE (*n* = 3). Means followed by ^*^indicate significant differences in relation to control (*P* = 0.05 by LSD procedure).

**Figure 2:**
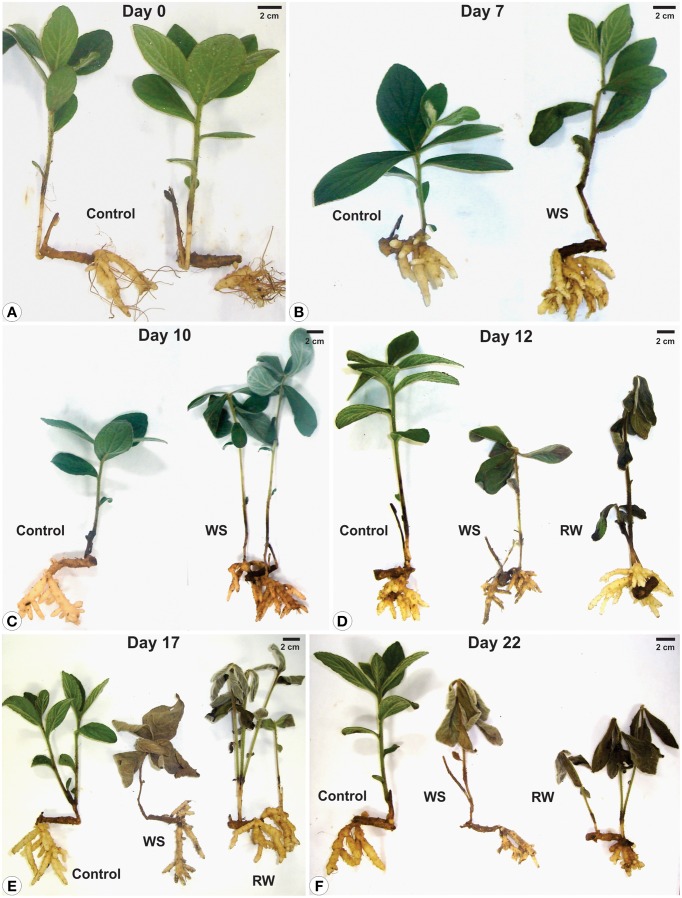
**Morphological aspects of plants (shoot and rhizophores) of ***Chrysolaena obovata*** submitted to daily watering—control, water suppression (WS) and re-watering (RW)**. Time zero **(A)**, day 7 **(B)**, day 10 **(C)**, day 12 **(D)**, day 17 **(E)**, day 22 **(F)**.

Soil moisture and water potential (Ψ_wsoil_) were unchanged in the control throughout the experiment. Water suppression (WS) caused a decrease in moisture, reaching 5% after 10 days, while re-watering (RW) led to soil moisture recovery in 2 days (Figures 1A,B). Similarly, water content in leaves and rhizophores were unchanged in control plants, diminished in WS and recovered in RW plants. In the latter, water content in aerial organs was only partially recovered (Figures 1C,D), while wilted aspect was maintained (Figure 2). WS treatment led to a decrease in water potential of leaves (Ψ_wleaf_) and rhizophores (Ψ_wrhiz_) (Figures 1E,F), followed by a recovery after re-watering at day 10. The soil water deficit imposed by WS treatment led to a severe and permanent wilting of the aerial organs from day 12 (Ψ_wleaf_ = −2.0 MPa), while re-watering at day 10 allowed the rhizophores (Ψ_wrhiz_ = −2.1 MPa) to recover the water potential within 2 days to levels similar to control plants (Ψ_wrhiz_ = −1.0 MPa). The rhizophores presented a turgid aspect in RW plants at days 12, 17, and 22 (Figures 2D,E,F), which paralleled the recovery of water content and water potential of these organs after re-watering. As shown in Figure 2, the aerial organs of RW plants maintained the wilted aspect as observed in WS plants, contrasting with the rhizophores of RW plants, that recovered the turgid aspect after re-watering at day 10 (Figures 2D,E,F). Figure 2 clearly shows that rhizophores of RW plants presented the same morphological aspect of those from DW plants (control).

### Structural analyses

**Figure 3:**
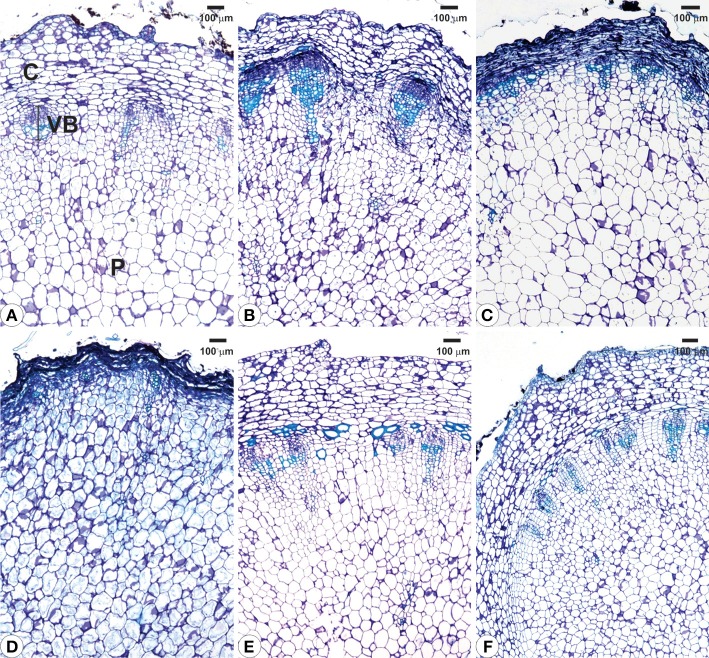
**Cross sections from rhizophores of ***Chrysolaena obovata*** submitted to daily watering—control (A), water suppression—day 10 (B), day 12 (C), day 22 (D), and re-watering—day 12 (E), day 22 (F)**. Note the beginning of anatomical changes in rhizophores **(B)**, flatness of outermost tissues **(C,D)** and structure restoration after re-watering **(E,F)**. C, cortex; P, pith; VB, vascular bundle.

**Figure 4:**
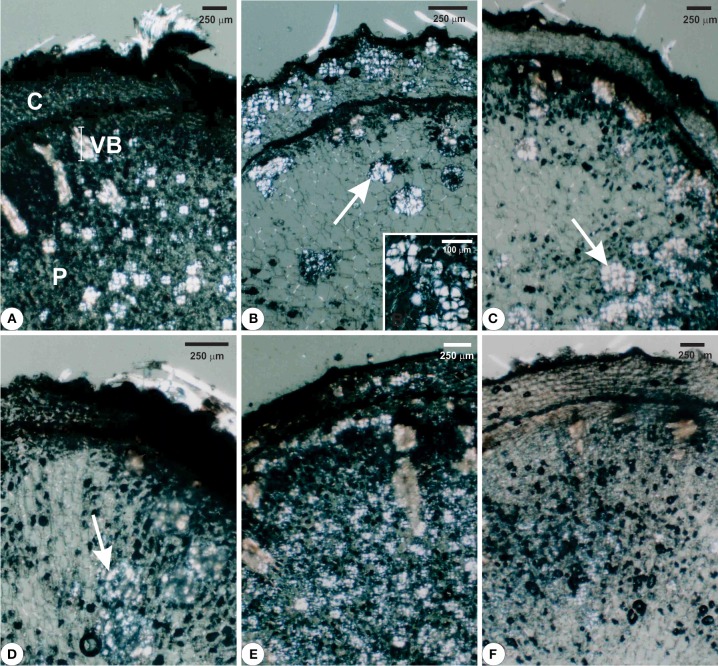
**Cross sections visualized under polarized light from rhizophores of ***Chrysolaena obovata*** submitted to daily watering—control (A), water suppression—day 10 (B), day 12 (C), day 22 (D), and re-watering—day 12 (E), day 22 (F)**. Note collapse of tissues from the outermost to the innermost region of the organ, mainly in the parenchyma of the cortex, secondary xylem and pith, highlighting the decrease of inulin sphero-crystals (**B–D**, arrows) and restoration of outermost tissues after re-watering **(E,F)**. Detail: **(B)** Cluster of inulin sphero-crystals. C, cortex; P, pith; VB, vascular bundle.

**Figure 5:**
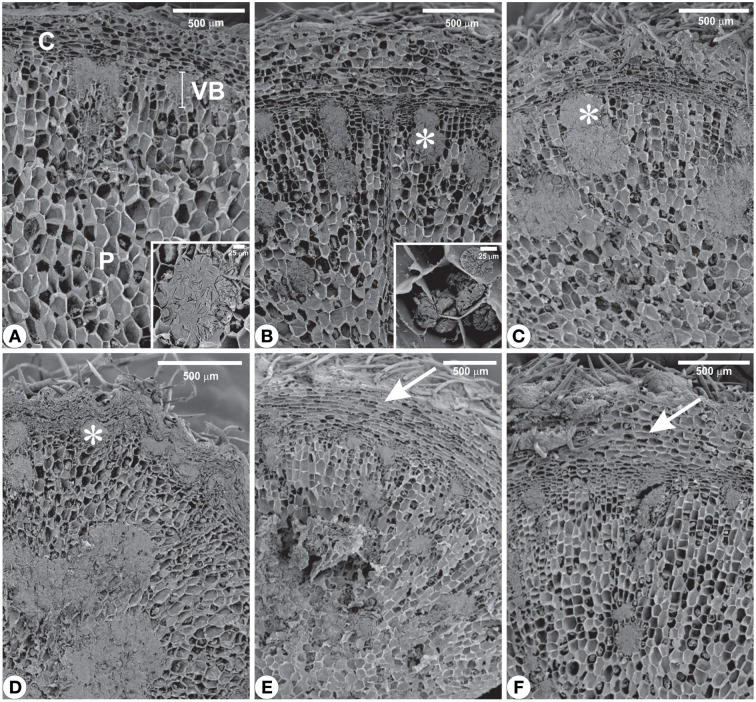
**Scanning electron microscopy from rhizophores of ***Chrysolaena obovata*** submitted to daily watering—control (A), water suppression—day 10 (B), day 12 (C), day 22 (D), and re-watering—day 12 (E), day 22 (F)**. Note the altered distribution of inulin (^*^), mainly after 22 days of water suppression **(D)**, and restoration of outermost tissues (arrows) after re-watering **(E,F)**. Details: **(A)** Cluster of inulin globules. **(B)** Isolated inulin globules. C, cortex; P, pith; VB, vascular bundle.

Structural analyses of the rhizophores (Figures 3, 4, 5) highlighted changes in plants subjected to water suppression and re-watering in relation to those daily watered (Figure 2). Rhizophores wilted in WS plants and recovered the initial morphological aspect after re-watering (Figures 2C,D,E,F). Structurally, rhizophores of control plants showed intact organization (Figure 3A) and the beginning of anatomical changes were visualized in WS plants collected at day 10 (Figure 3B), followed by progressive flatness of outermost tissues of the organ (Figures 3C,D) and structure restoration after re-watering (Figures 3E,F). Rhizophores of plants collected at day zero (control) presented a uniseriate epidermis with stomata and glandular and non-glandular trichomes. Sclereids were observed among the parenchymatic cells of the cortex. The endodermal cells were larger than the other cells of the cortical parenchyma. Vascular tissues are organized in bundles and the parenchymatic pith occupies the center of the rhizophore (Figure 3A). Rhizophores collected at 12 and 22 days from plants re-watered at day 10 (Figures 3E,F) showed anatomical characteristics similar to those of control plants (Figure 3A). Gradual changes in the structure of rhizophores were observed in plants undergoing 10, 12, and 22 days of water suppression (Figures 3B,C,D). At day 10, the cell walls of the epidermis, cortex and vascular tissues became sinuous, promoting cell flatness (Figure 3B). At day 12, this flatness was more evident since cell collapsing reached the outer layers of the pith (Figure 3C). At day 22 of WS, the epidermis, the cortex and the vascular tissues were completely collapsed, and the innermost cells of the pith featured sinuous walls (Figure 3D). The collapse of tissues from the outermost to the innermost region of the organ reduced tissues storing inulin sphaero-crystals (Figures 4C,D, 5C,D) which were restored after re-watering (Figures 4E,F, 5E,F). Inulin sphero-crystals were observed in the parenchyma cells of the cortex, vascular tissues and pith in control plants (Figures 4A, 5A), in WS plants after 10 days (Figures 4B, 5B), and in RW plants collected at 12 and 22 days after re-watering at day 10 (Figures 4E,F, 5E,F). Only rhizophores of WS plants showed a structurally compressed cortex at days 12 and 22 after water suppression, and a reduced amount of inulin sphero-crystals in this region (Figures 4C,D, 5C,D).

### Metabolic changes

**Figure 6:**
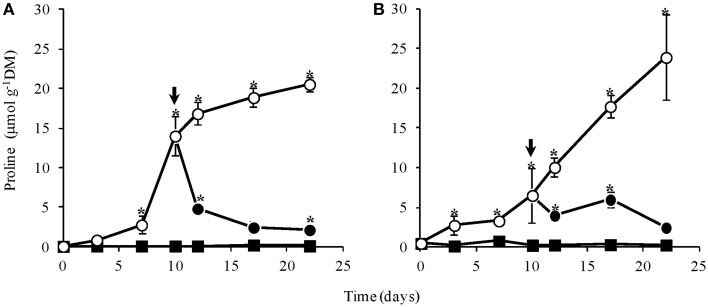
**Contents of proline in leaves (A) and rhizophores (B) of ***Chrysolaena obovata*** submitted to daily watering—control (■), water suppression (○) and re-watering (●)**. Arrows indicate re-watering at day 10. Values are means ± SE (*n* = 3). Means followed by ^*^indicate significant differences in relation to control (*P* = 0.05 by LSD procedure).

Proline contents in leaves and rhizophores of control plants were near zero throughout the experimental period while in WS plants, proline increased significantly after day 7 in aerial and from day 3 in underground organs, reaching values of approx. 20 and 25 μmol g^−1^ DM, respectively (Figure 6). In aerial organs, there was a marked increase until day 12, followed by a less pronounced increase, reaching maximum values at low leaf water content (Figure 1C). On the other hand, a linear increase was observed in rhizophores until the end of the experiment. Immediately after re-watering, the proline content declined in both, aerial organs (Figure 6A) and rhizophores (Figure 6B), and showed a tendency to reach values similar to the control at day 22.

**Figure 7:**
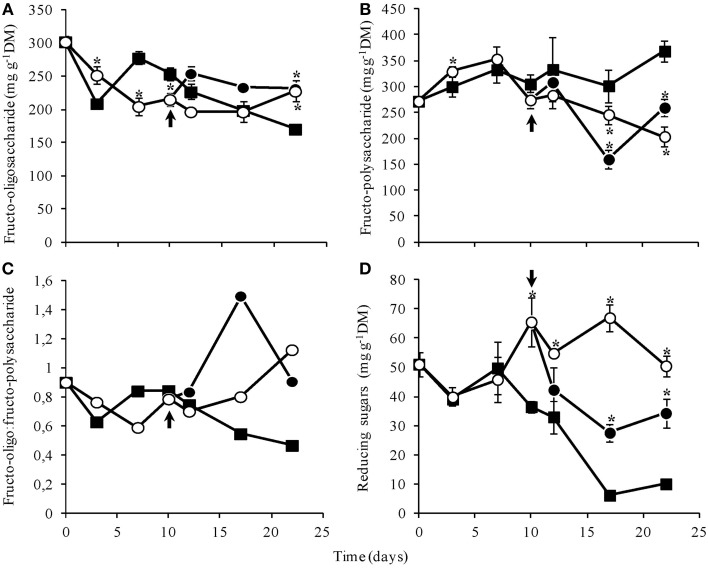
**Contents of fructo-oligosaccharides (A), fructo- polysaccharides (B), oligo:polysaccharide ratio (C), and reducing sugars (D) in rhizophores of ***Chrysolaena obovata*** submitted to daily watering—control (■), water suppression (○) and re-watering (●)**. Arrows in indicate re-watering at day 10. Values are means ± SE (*n* = 3). Means followed by ^*^indicate significant differences in relation to control (*P* = 0.05 by LSD procedure).

The amount of total fructans decreased slowly throughout the experiment, from 571.43 mg g^−1^DM at day 0 to 349.58 in control plants, 420.68 in WS plants and 450.97 mg g^−1^DM in RW, at day 22. At the end of the experimental period, fructo-oligosaccharide contents in WS and RW plants were significantly higher than in control (Figure 7A), while the polysaccharide contents were lower (Figure 7B). The ratio oligo:polysaccharides increased in WS plants from day 7, while RW plants showed a transitory increase at day 17, that was reverted at the end of the experimental period (Figure 7C). Water suppression caused a significant increase in reducing sugars from day 10 on and decreased rapidly after re-watering (Figure 7D). These results are consistent with the high proportion of hexoses, mainly fructose, as detected by anion exchange chromatography (HPAEC/PAD) (Supplementary Figure 1).

**Figure 8:**
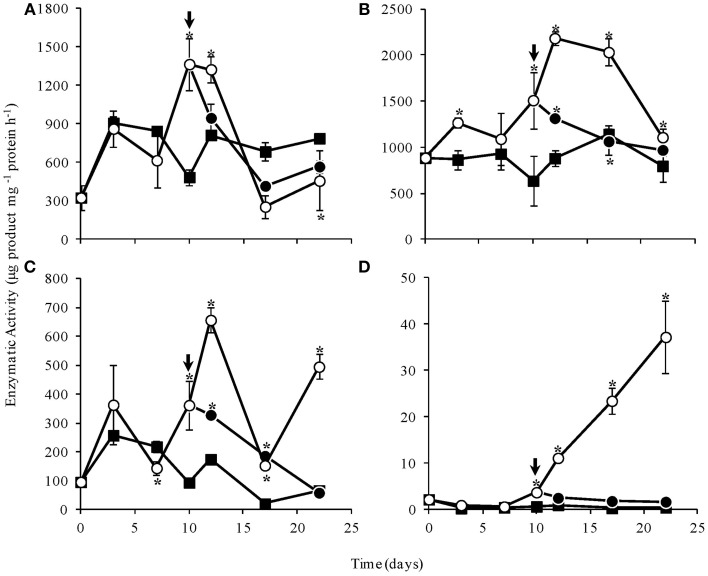
**Activities of 1-SST (A), 1-FFT (B), 1-FEH (C), and invertase (D) in rhizophores of ***Chrysolaena obovata*** submitted to daily watering—control (■), water suppression (○) and re-watering (●)**. Arrows indicate re-watering at day 10. Values are means ± SE (*n* = 3). Means followed by ^*^indicate significant differences in relation to control (*P* = 0.05 by LSD procedure).

1-SST activity in WS, as well as in control plants, increased initially, indicating fructan synthesis. At the end of the experimental period, 1-SST activity in WS plants was significantly lower than in control and RW plants (Figure 8A). A similar profile was observed for 1-FFT, mainly after day 10, although significantly high 1-FFT activity was observed in WS plants throughout most of the experimental period (Figure 8B). 1-FEH was in general significantly more active in WS plants, declining after re-watering and reaching low levels, close to those found in control plants, on day 22 (Figure 8C). Invertase activity increased notably in WS plants from day 10 on, and decreased after re-watering to values similar to control plants (Figure 8D). The marked increase in invertase activity in WS plants was consistent with the increase in reducing sugars and fructose, detected by HPAEC/PAD (Supplementary Figure 1).

## Discussion

Water suppression allowed a gradual development of water deficit in soil and in plants of *C. obovata*, as previously reported (Garcia et al., 2011), leading to pronounced structural and metabolic changes, including fructans and proline.

The localization of fructans in vascular tissues of underground reserve organs of Amaranthaceae (Vieira and Figueiredo-Ribeiro, 1993) and *Asteraceae* species from the Cerrado and rocky fields has been extensively reported (Isejima et al., 1991; Tertuliano and Figueiredo-Ribeiro, 1993; Melo-de-Pinna and Menezes, 2003; Vilhalva and Appezzato-da-Glória, 2006b; Appezzato-da-Glória and Cury, 2011; Vilhalva et al., 2011; Oliveira et al., 2013; Bombo et al., 2014; Joaquim et al., 2014; Silva et al., 2014, 2015). All these studies emphasized the relevance of fructans in protecting plants against drought and other environmental abiotic stresses in subtropical biomes (Carvalho et al., 2007).

In the present study, we showed that drought affected the content and distribution of inulin sphero-crystals in the rhizophores, mainly in parenchymatic cells of the cortex, vascular tissues and pith, accompanying early anatomical changes. In later stages of drought, the cortical and vascular tissues were completely collapsed, decreasing inulin accumulation in these tissues. Cellular collapse of covering tissue and outer cortical layers was also observed in rhizomes of *Costus arabicus*, a starch accumulating species, under water deficit. The degree of tissue damage was dependent on the intensity and duration of the water deficit (Costa et al., 2012). Additionally, Zhang et al. (2010) reported that water deficit could affect size distribution of starch granules in wheat cultivars. These authors showed that cultivars with high amylose content were less affected than those with low amylose content. This result demonstrates that starch grains studied by Zhang et al. (2010), similarly to inulin sphero-crystals, shown in the present work, are subjected to changes under water deficit. The understanding of how fructan concentration and compartmentalization, as well as how fructan molecule size distribution and structure are related to fructan crystallization requires further investigation using specific histochemical, chemical and physical methods, among others.

Structural changes in rhizophores of *C. obovata* were associated with physiological and metabolic responses to water suppression involving fructans, such as the increase in the ratio oligo:polysaccharides, mainly due to increase in 1-FEH activity stimulated by drought. This is consistent with the role of fructans in osmoregulation and drought tolerance, as evidenced for other species from tropical (Carvalho et al., 2007) and temperate regions (Valluru and Van den Ende, 2008). Plants of *C. obovata* under water deficit showed increased 1-FEH and invertase activities, such as reported for wheat (Virgona and Barlow, 1991; Yang et al., 2004), chicory (Van den Ende et al., 1998) and grasses (Spollen and Nelson, 1994; Clark et al., 2004). Increased 1-FEH activity also demonstrates its survival function under stressing conditions, as addressed previously (Van den Ende et al., 2001; Asega and Carvalho, 2004).

The increase in 1-FEH activity was accompanied by the increase in 1-FFT activity in plants of *C. obovata* under water deficit. As well known, 1-FFT acts in the redistribution of inulin chain sizes promoting the production of low degree of polymerization molecules, with higher osmoregulation properties and more susceptible to depolymerization by 1-FEH. The reversion of enzymatic activities after re-watering, to levels similar to those of control plants is consistent with the variation found in fructo-oligo:polysaccharides, and with glucose and mainly fructose levels.

Additionally, plants under water deficit showed increased proline contents, initially faster in leaves than in rhizophores, which further reached similar values at the end of the experimental period. Particularly in leaves, the accumulation of proline in *C. obovata* occurs even under low leaf water contents. The accumulation of osmoprotectors, including proline and fructans, is one of the mechanisms of adaptation to water stress. These molecules, which act in the osmotic balance, are accumulated in cells in response to drought stress and are degraded when water becomes available again (Tabaeizadeh, 1998). In fact significant correlations between proline in leaves and total fructans (*r* = −0.93, *P* = 0.007) and fructo-polysaccharides (*r* = −0.92, *P* = 0.01) were observed in WS treatment and between proline in leaves and total fructans (*r* = 0.91, *P* = 0.01) and fructo-oligosaccharides (*r* = 0.99, *P* = 0.0000005) in RW treatment. In rhizophores, proline also correlated inversely with total fructans (*r* = −0.97, *P* = 0.002) and fructo-polysaccharides (*r* = −0.94, *P* = 0.006) in WS, and positively with fructo-polysaccharides (*r* = 0.855, *P* = 0.03) in RW plants. Increasing of free-proline in leaves with very low water content was reported for nine desiccation tolerant species (Tymms and Gaff, 1979). More recently, Vander Willigen et al. (2004) also showed that desiccant-tolerate leaves of a resurrection grass, *Eragrostis nindensis* accumulate proline even under very low leaf relative water contents, suggesting that together others compatible solutes like sucrose, proline collaborates to a mechanical stabilization of the vegetative tissues in this species. Other functions suggested for proline are detoxification of reactive oxygen species and interaction with hydrophobic proteins. Furthermore, proline and other osmolytes can act as regulatory or signaling molecules, able to activate multiple responses in the process of adaptation to overcome environmental stresses (Maggio et al., 2002).

The contribution of this iminoacid in osmotic adjustment is also evidenced by the significant correlations with the decrease in water content (*r* = −0.92, *P* = 0.01) and water potential (*r* = −0.97, *P* = 0.002) of aerial organs and water content (*r* = −0.82, *P* = 0.05) and water potential in rhizophores (*r* = −0.996, *P* = 0.00002) of WS plants. However, the mechanism by which overproduction of proline contributes to osmoregulation in shoots of *C. obovata* remains to be elucidated, since, unlike in rhizophores, the high proline content in leaves did not contribute to avoid the process of shoot wilting that occurred at leaf water potential below −2.0 MPa (Figure 1). Different from shoot, the linear increase in proline contents observed in rhizophores suggests that in *C. obovata*, besides the osmotic role, this compound could also be an important source of carbon and nitrogen for the resumption of growth after re-watering, when proline pool decreases rapidly to values similar to those of well-watered plants, as pointed out for other species (Hare and Cress, 1997). Notwithstanding, changes in proline also correlated significantly with water content in rhizophores (*r* = −0.92, *P* = 0.009) and water potential of aerial organs (*r* = 0.91, *P* = 0.011) and rhizophores (*r* = −0.90, *P* = 0.014) of RW plants. In fact, proline involvement in recovery from stress is consistent with the observation of Handa et al. (1986) that the pool of proline in stressed tissues is usually followed by its rapid disappearance when the stress is removed.

Coupled with proline, the presence of fructans in rhizophores may have been the factor responsible for the decrease in water potential and maintenance of tissue turgor. Indeed, the proportion of oligosaccharides increased in plants under drought as evidenced by the increase in fructo-oligo:polysaccharides (*r* = −1.00, *P* = 0.000), and in the proportion of glucose (*r* = −0.85, *P* = 0.031) and fructose (*r* = −0.95, *P* = 0.003), final products of sucrose and inulin hydrolysis, negatively correlated with the decrease in rhizophore water potential. The involvement of these soluble compounds in osmoregulation mechanisms is also evidenced by their pronounced decrease in rhizophores immediately after re-watering, to levels similar to those observed in daily watered plants. Comparable to changes in proline and fructan metabolism, the typical rhizophore structure was also reestablished 2 days after re-irrigation (Figure 3E), indicating the reversibility and maintenance of the anatomical structure of the organ (Figure 3F).

Since *C. obovata* is seasonally exposed to winter drought (Rigui et al., 2015), changes in water status and in osmolytes such as soluble sugars and proline in water deficient plants clearly demonstrates the role of these compounds favoring survival in stressed environments such as the Cerrado. These compounds allow extensive osmoregulation under drought, an increasing worldwide phenomenon occurring also in subtropical regions, due to climate changes and other environmental constrains caused by growing anthropogenic interference.

### Conflict of interest statement

The authors declare that the research was conducted in the absence of any commercial or financial relationships that could be construed as a potential conflict of interest.
