# Supplementary material for: Structural and metabolic changes in rhizophores of the Cerrado species Chrysolaena obovata (Less.) Dematt. as influenced by drought and re-watering
Source: Front Plant Sci. 2015 Sep 17;6:721. doi: 10.3389/fpls.2015.00721 (PMC4585265; doi:10.3389/fpls.2015.00721)
Supplement: Supplementary file 1 [file Image1.PDF]

Supplementary Material

Structural and metabolic changes in rhizophores of the Cerrado species *Chrysolaena obovata* as influenced by drought and re-watering

Paola M. A. Garcia, Adriana H. Hayashi, Emerson A. Silva, Rita de Cássia L. Figueiredo-Ribeiro, Maria Angela M. Carvalho *

Correspondence: Corresponding Author: mam.carvalho@gmail.com

Supplementary Figure

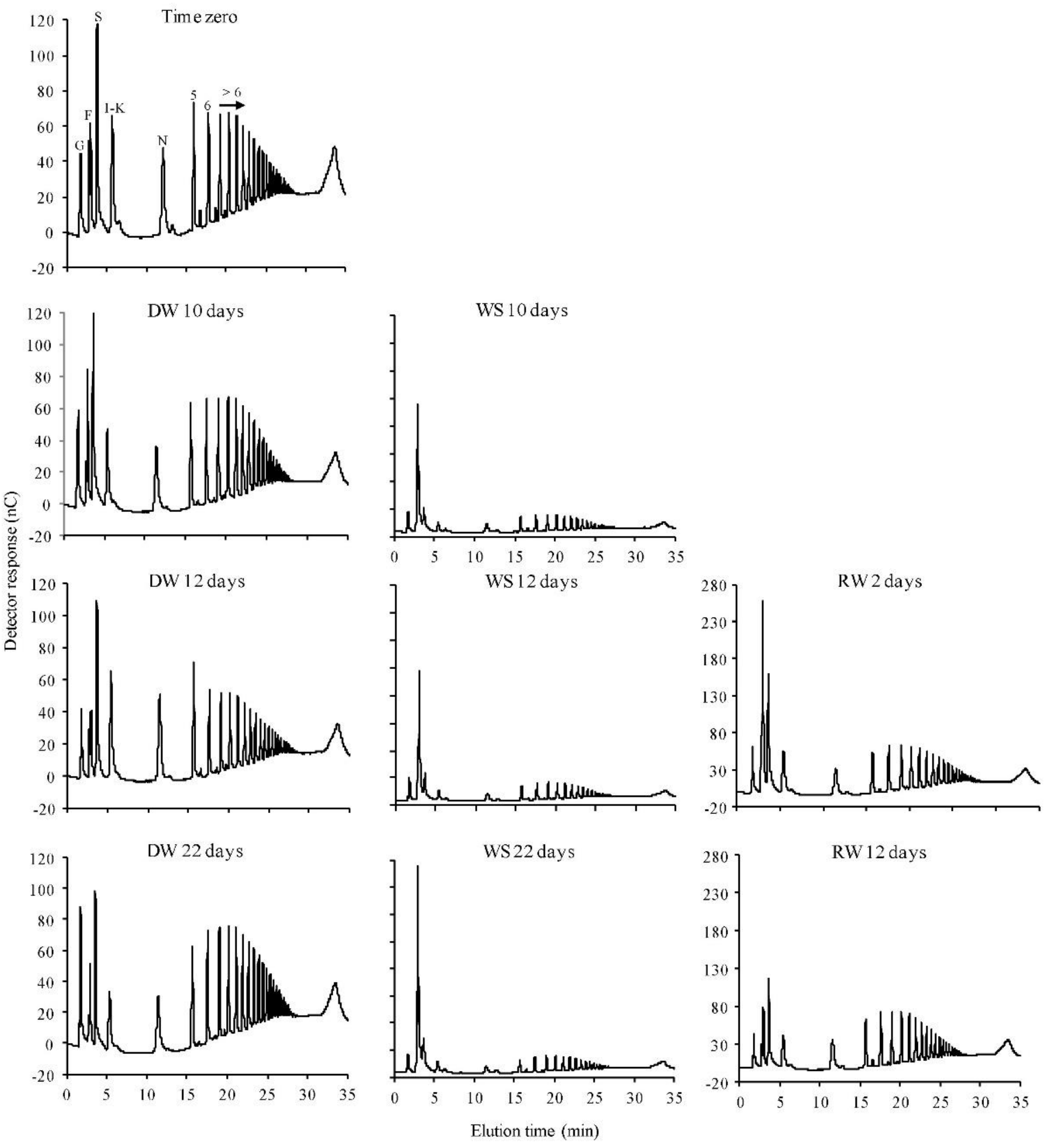

Supplementary Figure 1. HPAEC/PAD of fructo-oligosaccharides in rhizophores of *Chrysolaena obovata* submitted to daily watering - control (DW), water suppression (WS) and re-watering (RW). G - glucose, F - fructose, S - sucrose, 1-K - 1-kestose, N - nystose, >4: fructans with degree of polymerization higher than 4.
